# A Tool for Evaluating Medication Alerting Systems: Development and Initial Assessment

**DOI:** 10.2196/24022

**Published:** 2021-07-16

**Authors:** Wu Yi Zheng, Bethany Van Dort, Romaric Marcilly, Richard Day, Rosemary Burke, Sepehr Shakib, Young Ku, Hannah Reid-Anderson, Melissa Baysari

**Affiliations:** 1 Black Dog Institute Randwick, NSW Australia; 2 The University of Sydney Faculty of Medicine and Health, School of Medical Sciences Biomedical Informatics and Digital Health Sydney Australia; 3 Univ Lille, CHU Lille, ULR 2694 METRICS: Évaluation des Technologies de santé des Pratiques médicales Lille France; 4 INSERM, CHU Lille, CIC-IT/Evalab 1403 Centre d’Investigation Clinique Lille France; 5 University of New South Wales Randwick Australia; 6 Sydney Local Health District Sydney Australia; 7 Royal Adelaide Hospital Adelaide Australia; 8 Hunter New England Local Health District Newcastle Australia; 9 Macquarie University Hospital Sydney Australia

**Keywords:** medication alerts, decision support, human factors, assessment tool, usability flaws

## Abstract

**Background:**

It is well known that recommendations from electronic medication alerts are seldom accepted or acted on by users. Key factors affecting the effectiveness of medication alerts include system usability and alert design. Thus, human factors principles that apply knowledge of human capabilities and limitations are increasingly used in the design of health technology to improve the usability of systems.

**Objective:**

This study aims to evaluate a newly developed evidence-based self-assessment tool that allows the valid and reliable evaluation of computerized medication alerting systems. This tool was developed to be used by hospital staff with detailed knowledge of their hospital’s computerized provider order entry system and alerts to identify and address potential system deficiencies. In this initial assessment, we aim to determine whether the items in the tool can measure compliance of medication alerting systems with human factors principles of design, the tool can be consistently used by multiple users to assess the same system, and the items are easy to understand and perceived to be useful for assessing medication alerting systems.

**Methods:**

The Tool for Evaluating Medication Alerting Systems (TEMAS) was developed based on human factors design principles and consisted of 66 items. In total, 18 staff members recruited across 6 hospitals used the TEMAS to assess their medication alerting systems. Data collected from participant assessments were used to evaluate the validity, reliability, and usability of the TEMAS. Validity was assessed by comparing the results of the TEMAS with those of prior in-house evaluations. Reliability was measured using Krippendorff α to determine agreement among assessors. A 7-item survey was used to determine usability.

**Results:**

The participants reported mostly negative (n=8) and neutral (n=7) perceptions of alerts in their medication alerting system. However, the validity of the TEMAS could not be directly tested, as participants were unaware of any results from prior in-house evaluations. The reliability of the TEMAS, as measured by Krippendorff α, was low to moderate (range 0.26-0.46); however, participant feedback suggests that individuals’ knowledge of the system varied according to their professional background. In terms of usability, 61% (11/18) of participants reported that the TEMAS items were generally easy to understand; however, participants suggested the revision of 22 items to improve clarity.

**Conclusions:**

This initial assessment of the TEMAS allowed the identification of its components that required modification to improve usability and usefulness. It also revealed that for the TEMAS to be effective in facilitating a comprehensive assessment of a medication alerting system, it should be completed by a multidisciplinary team of hospital staff from both clinical and technical backgrounds to maximize their knowledge of systems.

## Introduction

### Background

Human factors is the scientific discipline that applies knowledge of human capabilities and limitations to improve the usability of systems, while reducing the potential for errors [1,2]. For decades, human factors research has been integral to the continuous improvement and innovation in industries outside of health care, such as aviation and automobile industries, with human performance limitations and human-system interactions taken into account when designing new technology [3-5]. For example, the failure to apply good human factors principles when designing aircraft and in-vehicle displays has been shown to lead to confusion and errors [3,6].

In recent years, the incorporation of human factors principles into the design of technology in health care has received increasing attention. Numerous studies have aimed to assess and improve clinical decision support in the form of electronic medication alerts [7-10], as it is well known that most recommendations from these alerts are not accepted or acted on by prescribers [11-14]. Excessive display of clinically irrelevant alerts can lead to alert fatigue, where important safety-critical information is ignored by clinicians (eg, doctors, pharmacists, and nurses) [14]. Studies have also investigated the factors influencing alert acceptance and found the following key factors affect the effectiveness of medication alerts: the usability of medication alerting systems, display of alerts, textual information included in alerts, and prioritization of alerts [15-21]. Furthermore, compared with poorly designed alerts, well-designed alerts using human factors principles resulted in faster work, fewer prescribing errors, less workload, and improved usability for prescribers [22,23].

However, what constitutes a well-designed medication safety alert and how compliance with human factors principles can be assessed and improved remain unclear. The Instrument for Evaluating Human Factors Principles in Medication-Related Decision Support Alerts (I-MeDeSA) was developed to evaluate compliance of drug-drug interaction alerts with human factors principles of design [10]. Comprising 26 items with binary scoring (ie, a score of 1 assigned to a yes response and 0 for a no response), the I-MeDeSA assesses compliance of electronic medication alerts with nine human factors principles of design, including alarm philosophy, placement, visibility, prioritization, color, learnability and confusability, text-based information, proximity of task components being displayed, and corrective actions [9]. Initially validated in the United States [10] and used in subsequent studies [7-9,24], several flaws with I-MeDeSA have been identified, including ambiguous item wording; arbitrary allocation of scores to human factors principles; and the need for more concrete definitions, clearer rationale for each item, and more explicit examples [7,8,24]. In our attempt to use I-MeDeSA to evaluate computerized alerts in Australian systems, we found many of the items to be irrelevant to Australian configurations [7], namely, items that assumed systems implemented more than one level of alert severity and multiple alert types. Thus, we set out to develop an evidence-based self-assessment tool that allows the valid and reliable evaluation of computerized medication alerting systems, in terms of their compliance with human factors principles. Our goal was to develop a tool that could be used by hospital staff with detailed knowledge of the hospital’s computerized provider order entry (CPOE) system and alerts (eg, a CPOE pharmacist who assisted in the building and configuration of the system) to identify and address deficient areas. This tool can also be used to facilitate the selection of the most user-friendly and functional medication alerting systems during the procurement process. With the increased adoption of digital health technology, a standardized tool using human factors principles to assess clinical decision support alerts, a crucial component of CPOE systems, would maximize alert acceptance and effectiveness and, therefore, broaden the potential safety benefits of medication-related alerts.

### Objectives

In this paper, we report the development of the Tool for Evaluating Medication Alerting Systems (TEMAS) and our initial attempts to assess its validity, reliability, and usability. In particular, we set out to determine whether (1) the items measure the compliance of medication alerting systems with human factors principles of design, (2) the tool can be consistently used by multiple users to assess the same system, and (3) the items are easy to understand and perceived to be useful for assessing medication alerting systems.

## Methods

### Development of the TEMAS

The pioneering work by Marcilly et al [25] identified 168 usability flaws related to general usability principles and medication-related alerting functions. A detailed description of each principle and its derivation can be found in a systematic qualitative review [25]. In summary, flaws specific to medication-related alerting functions were grouped into six categories, including low signal-to-noise ratio (eg, alerts are irrelevant or redundant), problems with alert content (eg, information required to make a decision is missing), nontransparency of alert functions (eg, no information on the alert severity scale), timing and display issues (eg, alert not displayed at the right moment to support decision-making), alert distribution issues (eg, alert not displayed to the right clinician), and problems with alert features (eg, no feature for reconsidering an alert later) [25]. Usability flaws were then matched with 58 design principles identified in the literature and two additional principles [26]. A usability flaw was matched with a design principle if it was in direct violation of the principle [26].

The TEMAS was developed by transforming each design principle into a checklist item, using usability flaws identified by Marcilly et al [25] to corroborate the accuracy of each item. Multimedia Appendix 1 includes some example design principles and their corresponding items in the TEMAS. Following this mapping process, the TEMAS consists of 66 items (Table 1), which fall into six meta-principles: (1) signal-to-noise ratio, (2) ability to support collaborative work, (3) ability to fit clinicians’ workflow and mental model, (4) display of relevant data within the alert, (5) transparency of system rules to the user, and (6) the inclusion of actionable tools within the alert. Each TEMAS item has two response options (ie, yes and no), with space provided for free-text comments. Before distributing the TEMAS to study participants, members of the research team, including experts in human factors, medication safety, digital health, and assessment tool development, checked and provided feedback on TEMAS items; however, pilot testing was not conducted with end users.

**Table 1:** Meta-principles assessed by the Tool for Evaluating Medication Alerting Systems (n=66).

Meta-principle	Items, n (%)	Example question
Optimize the signal-to-noise ratio	17 (26)	Does the alerting system use an evidence-based drug knowledge base to trigger alerts?
Support collaborative work	6 (9)	Does the alerting system trigger alerts to the appropriate team member (eg, medication administration alerts are triggered for nurses)?
Fit the clinicians’ workflow and mental model	16 (24)	Does the alerting system display alerts instantly (ie, no lag time)?
Display relevant data within the alert	10 (15)	Does the alert include information on the cause of the unsafe event (eg, medication name and dose)?
Ensure the system rules are transparent to the user	6 (9)	Does the alerting system inform users about the customization options available (eg, turning some alerts off)?
Include actionable tools within the alert	11 (17)	Does the alert provide a function for the user to modify an order?

### Participants and Study Sites

To identify potential participants for the initial evaluation of the TEMAS, a member of the research team at each study site nominated staff members at their hospital with relevant knowledge of their CPOE system and alerts (eg, a CPOE pharmacist responsible for maintaining the system). The study intended to recruit at least two participants from each site. Nominated staff members were contacted by email, and those who expressed an interest in taking part in the study were sent a participant information sheet and consent form. After submitting a signed participant information sheet and consent form, participants received a TEMAS pack. This pack included a copy of the TEMAS and a 7-item survey. Participants were asked to return completed TEMAS packs to the researchers via email or mail.

The study sites are presented in Table 2. In total, 18 participants across the 6 sites used the TEMAS to assess the medication alerting system at their hospital. Participants included pharmacists (n=11), clinical pharmacologists (n=2), nurses (n=2), doctors (n=2), and a business analyst. Participants were part of the CPOE system implementation team at their hospital or were responsible for maintaining or updating the system. On average, participants had 5.1 (SD 2.9) years of experience using their CPOE system, and as shown in Table 2, Cerner Powerchart and DXC Technology’s MedChart were the most frequently assessed systems.

**Table 2:** Study sites and number of participants (n=18).

Study site	Participants, n (%)	CPOE^a^ system in use
John Hunter Hospital (NSW^b^)	2 (11)	DXC Medchart
St Vincent’s Hospital, Sydney (NSW)	2 (11)	DXC Medchart
Macquarie University Hospital (NSW)	2 (11)	TrakCare
Concord Repatriation General Hospital (NSW)	5 (28)	Cerner Powerchart
Royal North Shore Hospital (NSW)	4 (22)	Cerner Powerchart
Queen Elizabeth Hospital (South Australia)	3 (17)	Sunrise EMR^c^

^a^CPOE: computerized provider order entry.

^b^NSW: New South Wales.

^c^EMR: electronic medical record.

### Study Design and Data Analysis

The evaluation consisted of assessing three components: the validity, reliability, and usability of the TEMAS. Participants were asked to independently use the TEMAS to evaluate the medication alerting system in use at their hospital and then complete a 7-item survey.

To assess validity, the survey included a free-text item on the perceived effectiveness of the alerts in the CPOE system and asked for supporting information or evidence with their response (eg, information on alert override rates, any formal or informal feedback received from users, and results from any in-house user surveys). Data collected from this item were analyzed by categorizing responses according to their positive or negative valence. Supporting information provided by participants was compared with TEMAS results to check whether the shortcomings of the alerting system identified by the TEMAS were consistent with those identified by in-house evaluations carried out by the hospitals.

To assess reliability, we compared the responses of participants working at the same hospital. Krippendorff α was calculated to determine interrater reliability.

**Figure 1:**
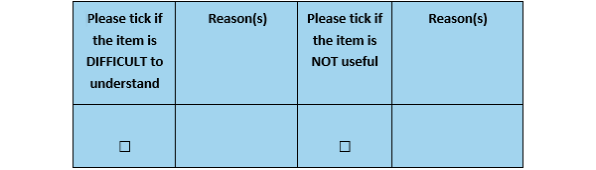
Feedback options for each Tool for Evaluating Medication Alerting Systems item to assess usability.

To assess usability, participants were given the opportunity to provide feedback on each TEMAS item to indicate whether an item was difficult to understand or was not useful (Figure 1). In addition, participants completed a usability survey (Multimedia Appendix 2), which collected basic demographic information, data on the ease of use using a five-point Likert scale (eg, item 1: I thought the TEMAS was easy to use), and free-text comments on the tool. The Likert-scale items were adapted from the system usability scale [27].

### Ethical Clearance

This study was approved by the Hunter New England Human Research Ethics Committee (reference no: HREC/18/HNE/237). In addition, research governance approval was obtained from each study site.

## Results

### Validity of the TEMAS

Participants gave mixed responses with regard to the perceived effectiveness of the alerts in their CPOE system. Of the 17 responses to this item (1 participant did not respond to this item), eight were negative, seven were neutral, and two were positive (Textbox 1).

**Selected comments of participants on the perceived effectiveness of alerts.**

**Positive**

- “I believe they’re reasonably effective, as they target the conditions that are ‘no-nos’” [Participant #1]
- “The alerts are coming from MIMS [Monthly Index of Medical Specialties] Australia and I believe their documentation is thorough.” [Participant #8]

**Neutral**

- “Somewhat effective. Pharmacists review quite a number of alerts via verification of medications, whilst there is a theoretical risk, there may not be many actual incidents.” [Participant #3]

**Negative**

- “Not very effective as prescribers have alert fatigue.” [Participant #2]
- “Poor; time consuming; click fatigue; alert fatigue; irrelevant alerts (e.g. non-current meds).” [Participant #6]
- “Too many alerts, hard to take out after we put in.” [Participant #7]

However, no participant provided evidence to support their personal assessment of alerts in their hospital’s system; that is, participants were unaware if their hospital collected meaningful data on the effectiveness of medication alerts in their CPOE system:

Most of the effect i.e. override rates etc. we don’t know

Participant #9

Has much room for improvement based on the evaluation factors in TEMAS however have no figures or paper to back it up

Participant #4

### Reliability

Table 3 presents Krippendorff α, which reflect interrater reliability among participants at each study site. To account for the missing data, α were also calculated for items with valid responses only (ie, a response of yes or no).

**Table 3:** Interrater reliability among participants from each study site (n=6).

Site	All responses, Krippendorff α (95% CI)	Valid responses, Krippendorff α (95% CI)
1	.30 (0.06-0.53)	.32 (0.07-0.53)
2	.46 (0.25-0.67)	.49 (0.27-0.68)
3	.39 (0.32-0.45)	.47 (0.39-0.55)
4	.26 (0.17-0.35)	.32 (0.21-0.42)
5	.40 (0.28-0.51)	.49 (0.37-0.62)
6	.38 (0.16-0.60)	.38 (0.16-0.60)

At the individual TEMAS item level, more than 10 items at 3 study sites did not receive a valid response from all participants working in those sites. They commented that they did not have the relevant knowledge to answer some items:

Not sure whether a doctor is able to make changes to the order and I’m not aware what their interface looks like.

Participant #4

Unsure - medical officer questions.

Participant #5

### Usability

Approximately 39% (7/18) of participants thought that the TEMAS was easy to use, with roughly 60% (3/5) of participants reporting that it was easy to understand. Approximately 41% (7/17) of participants found it to be a useful tool for identifying areas for improvement in their medication alerting system (Table 4).

**Table 4:** Usability of the Tool for Evaluating Medication Alerting Systems.

Survey item	Participants who selected strongly agree^a^ or agree^b^, n (%)	Participants who selected neutral^c^, n (%)	Participants who selected strongly disagree^d^ or disagree^e^, n (%)	Average score	Range (lower limit-upper limit)
I thought the TEMAS^f^ was easy to use. (n=18)	7 (39)	8 (44)	3 (17)	3.2	4 (1-5)
I thought the items in the TEMAS were easy to understand. (n=18)	11 (61)	4 (22)	3 (17)	3.5	3 (2-5)
I thought the TEMAS was useful in helping me to identify areas for improvement in my alerting system. (n=17)^g^	7 (41)	8 (47)	2 (12)	3.3	4 (1-5)

^a^Strongly agree was rated 5.

^b^Agree was rated 4.

^c^Neutral was rated 3.

^d^Disagree was rated 2.

^e^Strongly disagree was rated 1.

^f^TEMAS: Tool for Evaluating Medication Alerting Systems.

^g^One participant did not provide a response to this question.

Of the 66 TEMAS items, 33 (50%) were reported by at least one participant as difficult to understand due to item wording. However, only 15% (10/66) of items confused multiple participants (Table 5). Reasons provided by participants on why items were difficult to understand included a lack of clarity in the meaning of the item and their inability to provide a yes or no response (Table 5). Furthermore, 20% (13/66) of items were reported to be *not useful* by participants. However, only the item on whether the alerting system provided explanations on the classification of alert severity was deemed not useful by multiple participants (n=2 participants).

With regard to responses to free-text questions in the usability survey, participants provided additional comments on how the design of TEMAS could be improved, and other possible users of the tool:

All of the questions are yes/no, either it is or it isn’t - whereas in some cases it might be partially implemented. The questions are also worded such that a “no” answer to any question is a negative, and something should be done about it.

Participant #1

Think the target audience is unclear. Only some of these items can be optimised at a hospital level. Most of the issues are hard-coded and would need to be addressed by the vendor.

Participant #9

Needs to be amended as it’s unclear who i.e. IT people or clinical staff the TEMAS is aimed at. These groups require very different language

Participant #14

**Table 5:** Items in the Tool for Evaluating Medication Alerting Systems reported to be difficult to understand by multiple participants and example participant responses (n=18).

TEMAS^a^ item^b^	Participants, n (%)^c^	Example participant response
A4. Does the alerting system overcome missing data and reconcile multiple entries to trigger relevant alerts (eg, does the alerting system avoid using dated or unreliable data?)	6 (33)	“Extremely broad question” [Participant #10]
A9. Does the alerting system refrain from triggering an alert if a corrective action has already been taken?	5 (28)	“Not sure what ‘corrective action’ means” [Participant #9]
E6. Does the alerting system inform users of the unsafe events that are checked?	4 (22)	“What is an unsafe event, and where would this be defined?” [Participant #11]
A3. Does the alerting system use multiple sources (eg, patient record, laboratory result repository, and pharmacy) to trigger alerts?	3 (17)	“I am not sure what this is asking” [Participant #7]
A13. Does the alerting system group multiple recommendations for patients with comorbidities?	3 (17)	“Don’t think our system has this capability” [Participant #11]
A12. Does the alerting system prioritize alerts according to severity?	2 (11)	“This depends on what you mean by ‘prioritise’” [Participant #11]
D1. Does the alert include information on the cause of the unsafe event (eg, medication name and dose)?	2 (11)	“Unsure how to answer” [Participant #12]
D5. Does the alert include relevant patient information and provide a link for users to obtain further patient information?	2 (11)	“Example of patient info? Lab results?” [Participant #13]
F1. Does the alert provide a function for the user to modify an order?	2 (11)	“Only doctors can modify orders. Difficult for other professions to answer” [Participant #12]
F10. Does the alerting system allow users to remove alerts that are irrelevant or outdated?	2 (11)	“Difficult to classify as Y or N” [Participant #12]

^a^TEMAS: Tool for Evaluating Medication Alerting Systems.

^b^The letter and number preceding each item indicates section and item number, respectively.

^c^The values do not sum to 100% as they are not mutually exclusive.

## Discussion

### Principal Findings

In this study, we developed a self-assessment tool for medication alerting systems and aimed to evaluate the validity, reliability, and usability of the TEMAS; however, this proved difficult. The validity of the TEMAS could not be directly tested, as participants in the study were not aware of any in-house system evaluations carried out by the hospitals. As a result, participants reported that there was a lack of evaluation data to support their subjective assessment of the system. The reliability of the TEMAS, as measured by Krippendorff α, was low to moderate; however, feedback from users indicated that their knowledge of systems was highly variable. In terms of usability, according to the responses to a survey item, the majority of participants agreed that TEMAS items were easy to understand, although participants identified a number of items that needed improvement.

Several methods are used by hospitals to monitor and evaluate alert effectiveness, including the establishment of review committees consisting of pharmacists and doctors [28-30], development of visual analytic dashboards [13], and collection of end user feedback [31]. A key finding from this study was that no participating hospital had a systematic program in place to gather data on the effectiveness of medication alerts in their CPOE system. Although the view of participants on alerts in their systems were mostly negative, there was a lack of evaluation data to support these subjective assessments. Thus, the validity of the TEMAS could not be directly assessed. Future assessments of the TEMAS should consider applying a different participant screening process whereby only hospitals with available evaluation data are included. However, upon examining the TEMAS items considered to be *not useful* by study participants, only one item was deemed not useful by multiple users (n=2), suggesting that the content of the TEMAS was relevant in assessing medication alerting systems. Further evaluations of the TEMAS should be conducted in hospitals with in-house data on the effectiveness of alerts in their CPOE system.

Less than half of the participants indicated that the TEMAS was easy to use (7/18, 39%) and useful in identifying areas in the system for improvement (7/17, 41%), with more participants selecting *neutral* for these survey questions. This likely reflects that some TEMAS items needed improvement, which prevented respondents from fully endorsing the usability of the TEMAS. In response to the feedback received on individual TEMAS items, 33% (22/66) of items were modified to improve clarity and reduce ambiguities. To avoid confusion and misunderstanding due to the use of unsuitable terms (eg, corrective action, item A9; Table 6) and poor item wording (eg, item D1, Table 6), edits were made to the original TEMAS, taking into account participant comments on why they were unable to provide a response (eg, “I am not sure what this is asking” [Participant #5]). We also included examples to provide further clarification of the meaning of each item (Table 6). In response to feedback on difficulties in selecting a yes or no response for some items (eg, only some alerts provide clinically appropriate recommendations and suggest alternatives), the revised version of the TEMAS (Multimedia Appendix 3) included *partial* as an additional response option for each item. In addition, a note has been included to advise users that, depending on the local context, a response of *no* or *partial* to TEMAS items does not automatically indicate a weakness in the system.

**Table 6:** Examples of the revised Tool for Evaluating Medication Alerting Systems items.

Original item^a^	Revised item	Example to clarify the meaning of the item
A9. Does the alerting system refrain from triggering an alert if a corrective action has already been taken?	Does the alerting system refrain from triggering more alerts if the alert recommendation has already been followed?	The system refrains from triggering an alert if drug monitoring actions are already in place.
D1. Does the alert include information on the cause of the unsafe event (eg, medication name and dose)?	Does the alert include information on why the alert was triggered?	Medication names, dosages, and severity of interactions are included in drug-drug interaction alerts.
E6. Does the alerting system inform users of the unsafe events that are checked?	Does the alerting system inform users of the types of orders that will trigger alerts?	Clicking on a “more information” link in the help page informs the user that both order sentences and free-text orders can trigger alerts.

^a^The letter and number preceding each item indicates section and item number, respectively.

The reliability of the TEMAS was shown to be poor, likely reflecting the different levels of system knowledge possessed by the participants. Recruiting participants with equivalent, in-depth knowledge of their hospital’s medication alerting system proved difficult. Usually, one staff member possessed extensive knowledge of the hospital’s system (eg, a CPOE pharmacist), whereas other staff members within the same organization had more specialized knowledge of the hospital’s system (eg, a medical officer or clinical pharmacist). It may be that reliability was affected by differences in clinical practice settings, where staff members from different specialties use different functions of the system and have different views and understanding of the system based on their everyday use. Responses received from users suggest that the TEMAS may be more appropriately used by a team instead of an individual. For example, a participant in a pharmacist role was unsure of items related to prescribing medications, thus deferring these items to medical officers. There was also a suggestion to include system vendors in the evaluation process as “most of the issues are hard-coded and would need to be addressed by the vendor” [Participant #9]. Thus, evaluations carried out by a team consisting of representatives of system users from all clinical backgrounds would allow a more comprehensive evaluation of the alerting system. During this process, different parts of the TEMAS could initially be assigned to different team members based on their role and relevant expertise in the hospital (eg, prescribers are assigned to the *fit the clinician’s workflow and mental model* section).

The TEMAS is not dissimilar to a heuristic evaluation, which is a usability inspection method driven by experts to assess a design or product’s usability [32]. In heuristic analysis, a number of usability experts typically conduct an independent assessment of a product or interface and note usability violations, which are then amalgamated into a master list of usability problems. Using this approach, the identification of usability violations is highly dependent on the expertise of raters, with human factors or usability expertise associated with higher number of violations being detected. This is in contrast to the TEMAS, where we suggest users work as a team, not independently, to complete their alert assessment. This is because items cover a range of system aspects that are unlikely to be known to a single individual. We also suggest that the completion of the TEMAS should not be limited to usability experts but rather a multidisciplinary team of hospital end users (eg, pharmacists, doctors, nurses, and information technology professionals), each contributing their unique knowledge in the evaluation of a medication alerting system.

### Limitations

Our initial evaluation of the TEMAS had several limitations. First, we experienced difficulties in recruiting participants with in-depth knowledge of their hospital’s medication alerting system. Knowledge of some participants was role specific, limiting their capacity to complete the TEMAS, which impacted the interrater reliability. Future assessments of the TEMAS could use a team of system experts with varying expertise from different professional backgrounds. Second, we did not recruit a site with in-house evaluation data of their medication alerting system, thus limiting our ability to assess the validity of the TEMAS. As a result, findings derived from using the TEMAS to assess the strengths and weaknesses of medication alerting systems should be interpreted with caution and within the context of the organization. Furthermore, the TEMAS is designed to assess medication alerting systems in inpatient care and is likely to require some modification if it is to be used in other settings, such as pharmacy or outpatient settings. Finally, the TEMAS was not piloted with prospective end users before distribution to study sites; however, research team members with expertise in human factors, medication safety, and digital health checked and provided feedback on TEMAS items.

### Conclusions

On basis of the usability flaws matched to human factors design principles, the TEMAS was developed for hospitals to self-assess medication alerts in their CPOE system with the goal of improving the effectiveness of these alerts. This initial evaluation allowed the identification of components of the TEMAS that required modification to improve usability and usefulness, leading to changes to items and the addition of examples and a response option. To be effective in facilitating a comprehensive evaluation, we found that the TEMAS should be completed by a team of multidisciplinary hospital staff from both clinical and technical backgrounds. This study was integral to the evolution of the TEMAS and established a revised version ready for use. As a next step, the updated TEMAS will be trialed by teams of users to assess their medication alerting systems and to compare the assessment results of the TEMAS with the I-MeDeSA.

## Appendix

Multimedia Appendix 1 Design principles and corresponding Tool for Evaluating Medication Alerting Systems items.

Multimedia Appendix 2 Usability survey.

Multimedia Appendix 3 The Tool for Evaluating Medication Alerting Systems (revised).

## Abbreviations

CPOE: computerized provider order entry
I-MeDeSA: Instrument for Evaluating Human Factors Principles in Medication-Related Decision Support Alerts
TEMAS: Tool for Evaluating Medication Alerting Systems
