# Supplementary material for: A Tool for Evaluating Medication Alerting Systems: Development and Initial Assessment
Source: JMIR Med Inform. 2021 Jul 16;9(7):e24022. doi: 10.2196/24022 (PMC8325080; doi:10.2196/24022)
Supplement: Multimedia Appendix 1 [file medinform_v9i7e24022_app1.docx]

# Supplementary material 1: Design principles and corresponding TEMAS item

| Design principles | Corresponding TEMAS item |
| --- | --- |
| Consider temporal dimensions | A5. Does the alerting system distinguish between current and future orders? |
| Provide functions to support team awareness of alert management | B3. Does the alerting system allow multiple team members (i.e. doctors, nurses and pharmacists) to view responses to alerts (e.g. override reasons)? |
| Display the alert at the appropriate time | C1. Does the alerting system trigger alerts at the appropriate stage in a clinician’s workflow (e.g. at order entry)? |
| Suggest but do not impose | D6. Does the alert provide clinically appropriate recommendations (e.g. monitoring) and suggest alternatives (i.e. drug, dose and frequency)? |
| Explanations on the grading systems | E3. Does the alerting system inform users of the severity levels in use? |
| Function to send the alert to another clinician | F6. Does the alerting system allow users to forward the alert to another clinician? |
