# Supplementary material for: A Tool for Evaluating Medication Alerting Systems: Development and Initial Assessment
Source: JMIR Med Inform. 2021 Jul 16;9(7):e24022. doi: 10.2196/24022 (PMC8325080; doi:10.2196/24022)
Supplement: Multimedia Appendix 2 [file medinform_v9i7e24022_app2.docx]

# Supplementary material 2: Usability Survey

**Usability Survey**

***Demographics***

Position/role: _______________________________________________________

Name of EMM system in use: __________________________________________

How long have you been using EMM systems: _____________________________

What type of alerts are implemented in your system (e.g. allergy alerts):

___________________________________________________________________

***Usability of the TEMAS***

1. **I thought the TEMAS was easy to use** (Please circle the appropriate number)

| Strongly  disagree | Disagree | Neutral | Agree | Strongly  agree |
| --- | --- | --- | --- | --- |
| 1 | 2 | 3 | 4 | 5 |

1. **Can you think of anything that made the TEMAS hard to use?**

____________________________________________________________________________________________________________________________________________________________________

1. **I thought the items in the TEMAS were easy to understand** (Please circle the appropriate number)

| Strongly  disagree | Disagree | Neutral | Agree | Strongly  agree |
| --- | --- | --- | --- | --- |
| 1 | 2 | 3 | 4 | 5 |

1. **I thought the TEMAS was useful in helping me to identify areas for improvement in my alerting system** (Please circle the appropriate number)

| Strongly  disagree | Disagree | Neutral | Agree | Strongly  agree |
| --- | --- | --- | --- | --- |
| 1 | 2 | 3 | 4 | 5 |

1. **Can you think of any ways to improve the TEMAS?**

____________________________________________________________________________________________________________________________________________________________________

1. **Do you have any additional comments regarding the TEMAS and its usability?**

____________________________________________________________________________________________________________________________________________________________________

1. **How effective do you believe the medication alerts are in your current EMM system?**

In your response please include any information or evidence to support your view. E.g. information on alert override rates, including the most frequently overridden alert type, any formal or informal feedback received from users, results from user surveys or findings from any other evaluations that have been done, and any reports or academic papers. Please attach additional pages if needed.

_____________________________________________________________________________________________________________________________________________________________________________________________________________________________________________________
