# Supplementary material for: A Tool for Evaluating Medication Alerting Systems: Development and Initial Assessment
Source: JMIR Med Inform. 2021 Jul 16;9(7):e24022. doi: 10.2196/24022 (PMC8325080; doi:10.2196/24022)
Supplement: Multimedia Appendix 3 [file medinform_v9i7e24022_app3.docx]

# Supplementary material 3: The TEMAS (revised)

**The Tool for Evaluating Medication Alerting Systems (TEMAS)**

***This checklist should be completed with input from all user groups (e.g. pharmacists, doctors, nurses) and relevant stakeholders with in-depth knowledge of the medication alerting system (e.g. system trainers, IT department). Consultation with system vendors may be required for some items.***

***Please note that depending on local context, a response of ‘no’ or ‘partial’ does not automatically indicate a weakness in the system.***

|  | **Checklist item** | **Example** | **Yes** | **No** | **Partial** | **Comments** |
| --- | --- | --- | --- | --- | --- | --- |
| Optimise the signal-to-noise ratio | | | | | | |
| 1. A1. | Does the alerting system use an evidence-based drug knowledge base to trigger alerts? | *The system uses the Multum drug database to trigger drug-drug interaction alerts.* |  |  |  |  |
| A2*.* | Is the alert knowledge base updated to reflect changes in policies and guidelines? | *The knowledge base is updated when new medications are added to the hospital formulary.* |  |  |  |  |
| A3. | Does the alerting system draw information from multiple sources to trigger alerts? | *The system draws information from patient records and laboratory results to trigger alerts.* |  |  |  |  |
| A4. | Does the alerting system overcome missing data and reconcile multiple entries to trigger relevant alerts? | *The alerting system uses allergy data from different sources for the same patient to trigger alerts* |  |  |  |  |
| A5. | Does the alerting system distinguish between past, current and future orders? | *The system only triggers drug-drug interaction alerts for orders that are active at the same time.* |  |  |  |  |
| A6. | Does the alerting system increase the severity of the alert if an appropriate action is not taken? | *The system increases the severity of an alert when a patient receives multiple administrations of the unsafe order.* |  |  |  |  |
| A7. | Does the alerting system consider the patient’s clinical context before triggering an alert? | *The system considers the patient’s demographics, history, co-therapy, and test results before triggering an alert.* |  |  |  |  |
| A8. | Does the alerting system consider all components of the order before triggering an alert? | *The system considers the medication dose, route, and duration before triggering an alert.* |  |  |  |  |
| A9. | Does the alerting system refrain from triggering more alerts if the alert recommendation has already been followed? | *The system refrains from triggering an alert if drug monitoring actions are already in place.* |  |  |  |  |
| A10. | Does the alerting system consider the provider’s role and specialty before triggering an alert? | *The system presents different alerts to a junior doctor and a senior consultant.* |  |  |  |  |
| A11. | Does the alerting system trigger alerts based on defined severity levels? | *The system triggers major, moderate, and minor severity alerts.* |  |  |  |  |
| A12. | Does the alerting system consider the impact of the unsafe event on the patient when determining if an alert is triggered? | *The system triggers an alert when the unsafe event has the potential to cause harm to the patient.* |  |  |  |  |
| A13. | Does the alerting system group multiple recommendations for patients with co-morbidities? | *Alerts triggered as a result of a patient’s diabetes are grouped together.* |  |  |  |  |
| A14. | Does the alerting system aggregate low severity alerts and present these in a single display? | *The system presents low severity alerts together on a single page.* |  |  |  |  |
| A15. | Are alert numbers, override rates, and override reasons reviewed by the organisation? | *Alert information captured by the system are reviewed by the hospital.* |  |  |  |  |
| A16. | Does the alerting system allow customisation of alerts to meet the needs of organisations, groups and individual users? | *The system allows the hospital to switch off drug-drug interaction alerts.* |  |  |  |  |
| A17. | Does the alerting system maintain existing customisation following an upgrade? | *Drug-drug interaction alerts continue to be switched off after a system upgrade.* |  |  |  |  |
| Support collaborative work | | | | | | |
| B1. | Does the alerting system allow multiple team members (i.e. doctors, nurses and pharmacists) to determine what alerts have been triggered? | *The system allows doctors, nurses and pharmacists to see triggered alerts for a patient.* |  |  |  |  |
| 1. B2. | Does the alerting system allow multiple team members (i.e. doctors, nurses and pharmacists) to identify which alerts have been responded to? | *The system allows doctors, nurses and pharmacists to see what response has been made to an alert.* |  |  |  |  |
| B3. | Does the alerting system allow multiple team members (i.e. doctors, nurses and pharmacists) to view responses to alerts? | *The system allows doctors, nurses and pharmacists to see the override reasons documented for alerts.* |  |  |  |  |
| B4. | Does the alerting system trigger alerts to the appropriate team member? | *The system triggers medication administration alerts for nurses.* |  |  |  |  |
| B5. | Does the alerting system display alert content differently for different users while maintaining core patient and alert information? | *The system presents more pharmacological data in alerts to pharmacists.* |  |  |  |  |
| B6. | Does the alerting system provide the option to view more details? | *The system provides a hyperlink to more information within alert screens.* |  |  |  |  |
| Fit the clinicians’ workflow and mental mode | | | | | | |
| C1. | Does the alerting system trigger alerts at the appropriate stage in a clinician’s workflow? | *The system triggers an alert at order entry.* |  |  |  |  |
| C2. | Does the alerting system display alerts instantly (i.e. no lag time)? | *The system triggers an alert during order entry.* |  |  |  |  |
| C3. | Does the alert contain all relevant information on a single page without the need for scrolling or accessing additional tabs? | *Relevant alert information fits on a single pop up screen.* |  |  |  |  |
| C4. | Do alerts appear in a central location, over the CPOE/EMR screen? | *Alerts pop up in the middle of the computer screen.* |  |  |  |  |
| C5. | Does the alerting system allow quick and easy responses to alerts? | *A single mouse click is required to respond to an alert.* |  |  |  |  |
| C6. | Are users promptly returned to the appropriate stage of workflow after making a response to the alert? | *After overridding an alert, the user is allowed to continue with the order.* |  |  |  |  |
| C7. | Are alerts of different severity distinguishable from one another? | *Alerts of different severity are presented using different colours and shapes.* |  |  |  |  |
| C8. | Are alerts of different type distinguishable from one another? | *Allergy alerts are presented in a different colour to drug-drug interaction alerts.* |  |  |  |  |
| C9. | Does the alerting system use interruptive alerts only for warnings of high severity? | *A pop up alert is triggered for duplication of controlled medications, but not for allergies that result in a minor rash.* |  |  |  |  |
| C10. | Does the alerting system require users to enter an override reason for warnings of high severity? | *A reason must be documented when overriding a drug-drug interaction alert warning of the interaction between warfarin and ibuprofen.* |  |  |  |  |
| C11. | Is alert content structured in the same way in all alerts (i.e. does the alert content appear in the same location within an alert)? | *The severity of the alert is followed by the warning message.* |  |  |  |  |
| C12. | Is the alert content concise? | *An alert presents only the most relevant information.* |  |  |  |  |
| C13. | Does the alerting system present the most critical information at the top of the alert? | *A drug-drug interaction alert presents the severity level and the interacting drugs at the top of the alert.* |  |  |  |  |
| C14*.* | Does the alerting system present less critical information on demand? | *Mechanism of interaction in a drug-drug interaction alert is accessed via a hyperlink.* |  |  |  |  |
| C15. | Does the alerting system use consistent terms, phrases, classifications, colours and definitions in all alerts? | *An allergy alert is always classified as severe, major, moderate or minor, and the warning message is always in red.* |  |  |  |  |
| C16. | Does the alerting system use understandable and unambiguous text in all alerts? | *A drug-drug interaction alert clearly states which drugs are interacting and the potential consequences of the interaction.* |  |  |  |  |
| Display relevant data within the alert | | | | | | |
| D1. | Does the alert include information on why the alert was triggered? | *Medication names, dosages, and severity of interactions are included in drug-drug interaction alerts.* |  |  |  |  |
| D2*.* | Does the alert include information on the nature of the unsafe event and its likelihood? | *The drug-drug interaction alert explains that the interaction is highly likely to cause respiratory depression.* |  |  |  |  |
| D3. | Does the alert use colour and a signal word to indicate severity of the unsafe event? | *High severity alerts are always in red and include the word ‘warning’.* |  |  |  |  |
| D4. | Does the alert include information on the mechanism of the unsafe event? This should be presented on demand. | *Mechanism of interactions in a drug-drug interaction alert is accessed via a hyperlink.* |  |  |  |  |
| D5. | Does the alert include relevant patient information and provide a link for users to obtain further patient information? | *Patient lab results can be accessed by clicking on a link in the alert.* |  |  |  |  |
| D6. | Does the alert provide clinically appropriate recommendations and suggest alternatives (i.e. drug, dose and frequency)? | *For two medications that may interact and lead to hypokalaemia, the alert recommends close monitoring of potassium concentrations.* |  |  |  |  |
| D7. | In the case of multiple suggestions, does the alerting system display these in the order of importance? | *In an alert warning of a potential interaction between tacrolimus and diltiazem, the recommendation is to monitor the concentrations and effects (e.g. on renal function) of tacrolimus more frequently if diltiazem is started or stopped, and adjust the tacrolimus dose as necessary.* |  |  |  |  |
| D8. | Does the alert include links to references and guidelines? | *Links to mongraph and product information are included in a drug-drug interaction alert.* |  |  |  |  |
| D9. | Does the alerting system monitor whether alert recommendations are followed? | *The system checks to see if a dose reduction has been actioned following an overdose alert.* |  |  |  |  |
| D10. | Are users notifed if alert recommendations are not followed? | *The system notifies the doctor if a dose reduction has not been actioned.* |  |  |  |  |
| Ensure the system rules are transparent to the user | | | | | | |
| E1. | Does the alerting system inform users about the alerting algorithm/logic/formulas implemented within the system? | *Clicking on a ‘more information’ link in the help page provides information on the algorithms used to trigger alerts.* |  |  |  |  |
| E2. | Does the alerting system inform users about the customisation options available? | *Clicking on a ‘more information’ link in the help page provides information on how to turn off drug-drug interaction alerts.* |  |  |  |  |
| E3. | Does the alerting system inform users of the severity levels in use? | *Clicking on a ‘more information’ link in the help page informs the users that ‘contraindicated’ is the highest severity level of drug-drug interaction alerts triggered by the system.* |  |  |  |  |
| E4. | Does the alerting system provide an explanation to users on how severity is classified? | *Clicking on a ‘more information’ link in the help page explains why the interaction between allopurinol and azathioprine is classified as ‘contraindicated’.* |  |  |  |  |
| E5. | Does the alerting system inform users of what data are used to trigger alerts? | *Clicking on a ‘more information’ link in the help page informs the user that patient information and the Multum drug database are used to trigger drug-drug interaction alerts.* |  |  |  |  |
| E6. | Does the alerting system inform users of the types of orders that will trigger alerts? | *Clicking on a ‘more information’ link in the help page informs the user that both order sentences and free text orders can trigger alerts.* |  |  |  |  |
| Include actionable tools within the alert | | | | | | |
| F1. | Does the alert provide a function for the user to modify an order from within the alert? | *The user can modify the dose of a medication by clicking the ‘modify’ button within the alert.* |  |  |  |  |
| F2. | Does the alert provide a function for the user to discontinue the pre-existing order from within the alert? | *The user can discontinue a current order by clicking the ‘cancel pre-existing order’ button within the alert.* |  |  |  |  |
| F3*.* | Does the alert provide a function for the user to cancel the new order from within the alert? | *The user can cancel the order by clicking the ‘cancel new order’ button within the alert* |  |  |  |  |
| F4. | Does the alert provide a function for the user to order further tests or request monitoring of patients from within the alert? | *The user is able to request monitoring of the patient by clicking on a link within the alert.* |  |  |  |  |
| F5. | Does the alerting system allow users to delay an alert so that it can be actioned at a later time? | *The user is able to delay the triggering of an allergy alert for 24 hours so that the patient can be monitored before making a decision on whether to continue with the medication.* |  |  |  |  |
| F6. | Does the alerting system allow users to forward the alert to another clinician? | *A drug-drug interaction alert is forwarded from a pharmacist to the prescribing doctor.* |  |  |  |  |
| F7. | Does the alerting system allow alert content to be directly entered into patient records? | *The alert allows the user to navigate directly to the patient notes screen to record their decision to cancel a medication following an alert.* |  |  |  |  |
| F8 | Does the alerting system allow users to override the alert? | *The user is given an option to override the alert.* |  |  |  |  |
| F9. | Does the alerting system provide users with a list of override reasons to select from? | *The user selects ‘patient tolerates drug combination’ from a drop-down list after overriding a drug-drug interaction alert.* |  |  |  |  |
| F10. | Does the alerting system allow users to disable alerts for a particular patient if they are irrelevant or outdated? | *Allergy alerts to warn against prescribing penicillins are disabled for a patient who no longer exhibits signs of allergy.* |  |  |  |  |
| F11. | Does the alerting system allow users to update patient information from within the alert? | *The user changes a patient’s allergy status from within an allergy alert.* |  |  |  |  |

Please complete this table based on the assessment of the hospital’s medication alerting system. Please comment on the areas that need attention for each section of the TEMAS tool.

| **Section** | **Areas that need attention** |
| --- | --- |
| 1. Optimise the signal-to-noise ratio |  |
| 1. Support collaborative work |  |
| 1. Fit the clinicians’ workflow and mental model |  |
| 1. Display relevant data within the alert |  |
| 1. Ensure the system rules are transparent to the user |  |
| 1. Include actionable tools within the alert |  |
